# Exploring the equity impact of a maternal and newborn health intervention: a qualitative study of participatory women’s groups in rural South Asia and Africa

**DOI:** 10.1186/s12939-019-0957-7

**Published:** 2019-04-11

**Authors:** Joanna Morrison, David Osrin, Glyn Alcock, Kishwar Azad, Jyoti Bamjan, Bharat Budhathoki, Abdul Kuddus, Mahfuza Akter Mala, Dharma Manandhar, Albert Nkhata, Shrijana Pathak, Tambosi Phiri, Shibanand Rath, Prasanta Tripathy, Anthony Costello, Tanja A. J. Houweling

**Affiliations:** 1 Institute for Global Health, University College London, 30 Guilford Street, London, WC1N 1EH UK; 2 BADAS, Room No-390, BIRDEM Building 122,Kazi Nazrul Islam Avenue,Shahbagh, Dhaka, 1000 Bangladesh; 3 MIRA, PO Box 921, Thapathali, Kathmandu, Nepal; 4 ActionAid Bangladesh, R#136, H#08, Gulshan 1, Dhaka, 1212 Bangladesh; 5 MaiMwana Project, Box 2, Mchinji, Malawi; 6 Jharkhand, India; 7 Institute for Global Health, University College London, 30 Guilford Street, London, WC1N 1EH UK; 8 Rotterdam, Netherlands

**Keywords:** Socio-economic, Health inequalities, Community participation, Behavior change, Community mobilization, Maternal and child health, Qualitative research

## Abstract

**Background:**

A consensus is developing on interventions to improve newborn survival, but little is known about how to reduce socioeconomic inequalities in newborn mortality in low- and middle-income countries. Participatory learning and action (PLA) through women’s groups can improve newborn survival and home care practices equitably across socioeconomic strata, as shown in cluster randomised controlled trials. We conducted a qualitative study to understand the mechanisms that led to the equitable impact of the PLA approach across socioeconomic strata in four trial sites in India, Nepal, Bangladesh, and Malawi.

**Methods:**

We conducted 42 focus group discussions (FGDs) with women who had attended groups and women who had not attended, in poor and better-off communities. We also interviewed six better-off women and nine poor women who had delivered babies during the trials and had demonstrated recommended behaviours. We conducted 12 key informant interviews and five FGDs with women’s group facilitators and fieldworkers.

**Results:**

Women’s groups addressed a knowledge deficit in poor and better-off women. Women were engaged through visual learning and participatory tools, and learned from the facilitator and each other. Facilitators enabled inclusion of all socioeconomic strata, ensuring that strategies were low-cost and that discussions and advice were relevant. Groups provided a social support network that addressed some financial barriers to care and gave women the confidence to promote behaviour change. Information was disseminated through home visits and other strategies. The social process of learning and action, which led to increased knowledge, confidence to act, and acceptability of recommended practices, was key to ensuring behaviour change across social strata. These equitable effects were enabled by the accessibility, relevance, and engaging format of the intervention.

**Conclusions:**

Participatory learning and action led to increased knowledge, confidence to act, and acceptability of recommended practices. The equitable behavioural effects were facilitated by the accessibility, relevance, and engaging format of the intervention across socioeconomic groups, and by reaching-out to parts of the population usually not accessed. A PLA approach improved health behaviours across socioeconomic strata in rural communities, around issues for which there was a knowledge deficit and where simple changes could be made at home.

## Background

Progress in improving maternal and newborn health has been inadequate to meet development targets, and it is estimated that nearly 3 million newborn infants die each year, most of them in poor populations in low and middle income countries [1, 2]. Poor mothers and infants often do not receive interventions such as skilled birth assistance and essential newborn care [3–5]. Furthermore, better-off socioeconomic groups tend to benefit first and to a greater extent from newly introduced health and behavioural interventions [6]. Systematic reviews have shown that evidence on how to reduce socioeconomic inequalities in neonatal mortality within countries remains scant [7–9].

Participatory learning and action (PLA) through women’s groups has, in randomised controlled trials in South Asia and Africa, been shown to reduce newborn mortality by up to 33% and maternal mortality by up to 55%, provided that population coverage is sufficiently high [10]. The intervention consisted of local facilitators who convened women’s groups and led them through PLA cycles related to maternal and newborn health. The groups identified and prioritised problems associated with pregnancy, delivery, and the newborn period, and together with communities, they planned and implemented strategies to address these problems. Strategies included, for example, home visits to pregnant women who were not part of the groups to tell them about safe birthing practices and newborn care, the distribution of safe delivery kits, and stretcher schemes to bring women in labour to the hospital. The PLA approach was based on the theory that to transform society and address inequalities, local stakeholders should actively participate in identifying problems, planning, implementing and evaluating change processes or interventions [11, 12]. Through participation, a collective critical consciousness is developed. Interaction with those in a similar situation, who share beliefs and purpose, enables individuals and groups to become empowered to work toward change. Knowledge is developed through a process of action and reflection, stimulating change [13, 14] Process evaluation research has suggested that changing social norms and building community capacity were key mechanisms for behaviour change, but there was little engagement with larger power structures or policy makers. Change was often localised and incremental, but enough to save newborn lives [15–17].

This intervention has been shown to reach all socioeconomic groups equally [18] and a recent meta-analysis of randomised trials of PLA interventions found that the impact on neonatal mortality was at least as strong among the socioeconomically most marginalised as among better-off groups [19, 20]. The intervention generally had no impact on health care use. Conversely, substantial effects on maternal and newborn home care practices were observed in all socioeconomic strata. Exploring the mechanisms that led to improved home care practices across socioeconomic groups, among women’s group members and in the wider community provides policy relevant information about how to reduce inequalities in neonatal health and survival.

In this paper, we present findings from qualitative research with community members in the trial intervention areas to explore how positive behaviour change and improvements in newborn survival were stimulated across all socioeconomic strata. We discuss the key components of the intervention that underlie its success in improving newborn survival and healthy practices equitably and how these relate to behaviour change theories.

## Methods

### Settings

We collected data from four trial sites, in Bangladesh, India, Nepal, and Malawi. All the sites were rural, with populations mainly engaged in subsistence agriculture. Mortality rates were relatively high at all sites. In Bangladesh, the trial was conducted in Faridpur, Bogra, and Moulvibazaar districts [21]; in India, in Jharkhand and Orissa, two of India’s poorest states, with large tribal populations [22]; in Nepal, in the hilly district of Makwanpur, south of the capital Kathmandu [23]; and in Malawi, in Mchinji district in the central region [24].

The cultural context of study areas varied. In Bangladesh, 80% of the population were Muslim, and *purdah* (female seclusion) was routinely practised, restricting women’s freedom of movement and their access to public spaces [25]. In Nepal, 65% of the population were of marginalised Tamang Buddhist ethnicity and 17% were of more advantaged Hindu Brahmin Chhetri ethnicity [26]. In the Indian study site, 80% of the population were from scheduled castes or tribes [22]. In the Malawi study site, 95% were Christian and 88% were of Chewa ethnicity. 74% of women in Nepal and 75% of women delivering in study areas in India had never been to school, whereas in study sites in Bangladesh, 19%, and in Malawi 21%, had never been to school. Very few women in all study sites had 11 or more years of schooling. All studies were located in poor rural areas with low rates of institutional delivery. The Malawi site had the highest institutional delivery rate at 40% and Nepal had the lowest at 5%. Newborn mortality rates were high in all sites, with the highest rate in the Indian site at 57.9 per 1000 live births.

The women’s group interventions were similar in that they all used PLA cycles. Groups implemented locally relevant and feasible strategies such as making and selling clean home delivery kits, raising funds, awareness-raising, home visits to pregnant women and their families, and interactions with health facilities [15, 16, 27]. In the four trial sites, between 37 to 57% of pregnant women participated in groups in the final trial year. Socioeconomic differences in participation in the groups were small [18]. Further details about the intervention can be found in the trial papers [21–24].

### Study design

We based our design on quantitative findings about the equity impact of the intervention [20] and on the trials’ process evaluation research [15, 16, 27]. Potential sources of behaviour change were extracted from this process evaluation research. Researchers at the trial sites received summaries of the quantitative findings and a table of the potential sources of behaviour change from JM (first author) and TH (last author). Sources of behaviour change were identified as: developing knowledge; having the confidence to act; the characteristics and behaviour of the facilitator; having increased access to economic and other resources; dissemination of information to non-attenders; increased social acceptability of behaviour change; enhanced community capacity to deal with problems; development of social support; community readiness for change; and interaction with wider governance structures. JM, TH and researchers from all sites discussed the quantitative findings and prioritised topics to explore with community members through qualitative methods. In prioritising topics, we considered the extent to which they had been explored in previous studies [15–17] and their cross-site relevance. We also explored some topics that were not featured in the Table. A research protocol and topic guides were written in English by JM and discussed at each site before reaching consensus on the final protocol and topic guides. Both allowed for adaptation to context, but the focus and methods were similar which enabled comparative analysis and theoretical generalisability.

At each site, a senior trial team researcher recruited, trained, and managed qualitative investigators. At sites in Nepal and India, the senior researchers had been conducting process evaluation and were experienced in qualitative research. In Bangladesh and Malawi, an experienced qualitative researcher was recruited.

#### Study design in the Asian sites

The design was similar for trial sites in Bangladesh, India and Nepal. Research teams agreed that purposive sampling of poor and better-off participants was necessary, but adherence to strict inclusion criteria about socioeconomic status when purposively sampling participants could cause offense. The process of building rapport is key to having an open, honest discussion and this could be jeopardised if participants knew they were being approached because they were ‘poor’. Instead, participants were purposively sampled from three trial intervention clusters that included locally defined poor and better-off areas. Local research team members and health volunteers categorised areas and located participants according to sampling criteria (Table 1). In each cluster, we conducted a key informant interview (KII) with a women’s group facilitator, a focus group discussion (FGD) with women who had attended the group in a poor area, and a FGD with women who had not attended the group in a similar area. This enabled us to explore the effect of the intervention in the general community, as well as within group attenders. Women who had attended a group were asked to identify a woman who had been pregnant during the trial whom they felt had been helped by the intervention. We then conducted a semi-structured interview (SSI) with her. The same data collection occurred in better-off areas in two of the three sampled clusters at each site. We collected more data in poor areas because we felt it was important to understand the experience of the intervention from poor women’s perspectives. Researchers took informed verbal consent from participants and no-one refused to participate.

**Table 1:** Data collection at all sites

Stakeholder	Method	Number
Key informant (women’s group facilitator)	KII	12
Group attenders (better-off areas)	FGD	7
Group attenders (poor areas)	FGD	10
Group non-attenders (better-off areas)	FGD	8
Group non-attenders (poor areas)	FGD	11
Women who showed good care behaviour (better-off areas)^a^	SSI	6
Women who showed good care behaviour (poor areas)^a^	SSI	9
Facilitation supervisors^b^	FGD	1
Facilitators^b^	FGD	1
Monitoring supervisors^b^	FGD	1
Enumerators^b^	FGD	1
Facilitators and enumerators^b^	FGD	1
Total		68

*KII* Key informant interview, *FGD* Focus Group Discussion, *SSI* Semi Structured Interview

^a^Bangladesh, India, Nepal

^b^Malawi

Researchers used topic guides in interviews and discussions, translated from English and adapted to the local context. These were split into three sections. The first section invited respondents to remember the time and context of the women’s group intervention and discuss specific examples of women who had been pregnant and given birth, including their behaviour and how they were affected by the women’s group. The first section of the topic guide for the SSI asked the woman to discuss her own experience and how she felt the group had affected or not affected her and her family’s behaviour. The second section explored specific care behaviours that we had seen improve, and how poor women and families were particularly enabled to change their behaviour. This allowed site-specific exploration of issues - for example, in India, participants discussed the reasons why poor women showed increased uptake of iron supplementation. The third section summarised the discussion, and invited participants to prioritise how women’s groups had enabled behaviour change, and how they might have particularly affected poor women and families. Prioritisation used participatory voting, putting pebbles on meta-cards which had a short description and symbol for each topic. Emergent topics were added to meta-cards by researchers. Topics covered dissemination and discussion of ideas and knowledge, acceptability of home care behaviours, and community capacity and social support.

#### Study design in Malawi

In Malawi, the intervention had a similar mortality effect on poor and better-off families, but had no effect on home care practices and health care uptake in either socioeconomic group. To understand these findings, we purposively sampled research team members who had been involved in the trial intervention and monitoring, as well as community and group members. We explored how interventions and monitoring systems might have affected the mortality impacts and reporting of behaviours. The proximity of trial team members to communities throughout the intervention made them key informants. We conducted one FGD with facilitation supervisors, one with monitoring supervisors, one with female group facilitators, one with male trial enumerators from control areas, and one with a mixed gender group of facilitators and trial enumerators. In the community, we categorised areas as previously described, and conducted two FGDs with women’s group attenders from poor and better-off households, and two FGDs with women from poor and better-off households who had not attended the groups. We also conducted four KIIs with community members, four with trial researchers, and four with facilitators in two clusters. A senior qualitative researcher who was an established MaiMwana team member led discussions. This researcher had not been involved in the trial. We took informed verbal consent from participants and no-one refused to participate. Topic guides were developed in English in discussion with JM, and subsequently translated. We explored how the activities and strategies of the women’s groups might have been effective in reducing neonatal mortality among both the poor and better-off.

### Data management and analysis

Data were digitally recorded, transcribed in the local language, and analysed at each site by field and senior researchers according to a coding structure. This coding structure was developed by JM and discussed with researchers at each site. Codes were based on topic guides and researchers were encouraged to add emergent codes. Analysis was conducted at each research site by researchers using techniques of manual analysis (by printing transcripts, cutting and categorising), highlighting text in different colours in Microsoft Word, or in Nvivo version 10. Researchers used a modified framework approach [28]. They tabulated a description of each code in English and sought to triangulate findings among different respondent types and clusters. They wrote descriptions of any variation or similarities in the data, extracted relevant quotes in English, and wrote a description of how the code related to others. Researchers then wrote several paragraphs in response to the research questions: Why were improvements in behaviour similar among low and high socioeconomic groups? To what extent did community capacity and social support, dissemination and spread of ideas, increased access to resources (clean delivery kits, in particular), and increased acceptability of behaviours explain the impact of the women’s groups? A sample of representative transcripts were translated into English and sent to JM. She coded these data independently, and wrote descriptions of the main findings in order to test the validity of the findings through ‘peer examination’ [29]. She then reviewed the tables, transcripts and answers to research questions and compared responses across sites. A description of the common themes was given to researchers who considered their completeness and validity.

## Results

**Fig. 1:**
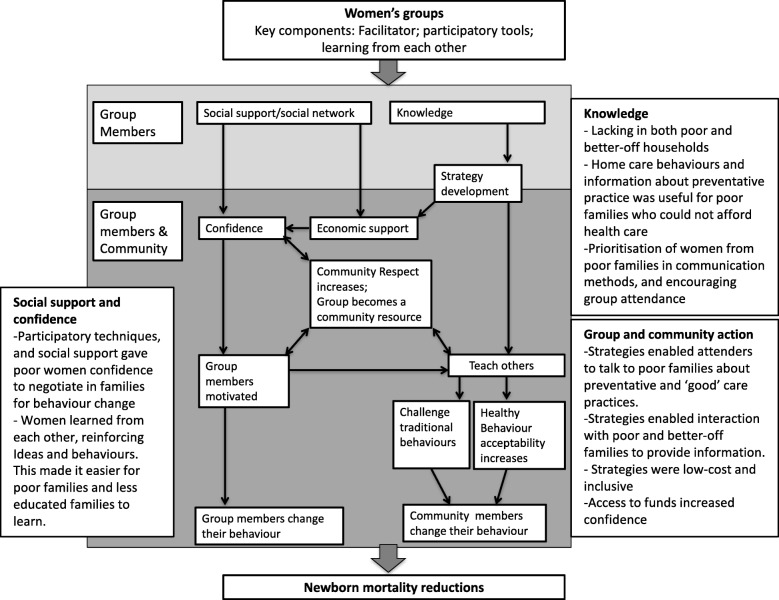
Mechanisms of equitable behaviour change

Figure 1 summarizes the findings regarding mechanisms through which the women’s groups led to equitable behaviour change. The process of learning and developing knowledge, the social support gained through group participation, and the process of taking action as a group were key mechanisms identified in this study.

### Knowledge, dissemination, and spread of ideas

Respondents from all sites said that both women and community members attending and not attending the groups learned from the intervention. They gained knowledge about how to prevent and manage maternal and newborn health problems, and could therefore take informed action.

#### Learning in the groups

Group attenders learned how to care for pregnant women, prepare for a delivery, and care for mothers and infants during delivery and postpartum. This included care to prevent and treat illness: “In the women’s group we learned that after the child enters the womb you should go for a check-up straight away, and then once in the seventh month, then in the eighth and ninth months. You need to go for a check-up four times to see whether the child is all right or not, to see whether it is healthy or not. Then you get weighed. See! We’ve learnt all of these things!” (Bangladesh, attender, poor, FGD). Participants at all sites discussed how other non-governmental organisations and government and media campaigns had been useful in disseminating information and increasing knowledge, but some felt that the group enabled a more engaging form of learning: “(community members) also get all this information from the radio and television, but the (women’s group) meeting is giving something that we do not get anywhere else. Even if tribal women watch TV, they do not understand in detail, but they get knowledge about health through this meeting. (The facilitator) sits with us and makes us understand.” (India, attender, poor, changed behaviour, SSI). In Bangladesh, women’s group attenders reported that traditional birth attendants (TBAs) also learned from the group.

#### Relevance for poor and better-off women

Some participants felt that both poor and better-off women held traditional beliefs and lacked knowledge about healthy behaviours, and that community members from all socioeconomic strata were able to learn and be convinced by the intervention. Group attenders and non-attenders emphasised that knowledge was useful for all socioeconomic strata: “Rich families have money, but that doesn’t mean to say they know everything. Money is not worth anything if there is a lack of education or ideas. If there was no women’s group then how would better-off women and poor families know about good home care behaviour?” (Nepal, non-attender, poor, changed behaviour, SSI). At the same time, knowledge provided by the groups on how to prevent problems was considered particularly relevant for poor women. While better-off women could go to a private clinic for delivery or treatment, preventing health problems was more important for poor women: “People from poor communities are getting more benefit in comparison to the better-off because the better-off are able to go to hospital… poor people depend on and benefit from the meetings more than the better-off. The better-off have other options.” (India, non-attender, poor, changed behaviour, SSI). Recommendations for low-cost home care behaviours to prevent illness were particularly well received by poor families.

Facilitators in Malawi said that messages were “shared equally” among better-off and poor families and that there was no discrimination in implementing strategies, visiting homes of non-attenders or having discussions. Groups in Nepal and India had focused on poor families, while not neglecting the better-off: “Group attenders gave priority to poor families and counselled them that good care behaviours were important.” (Nepal, non-attender, poor, FGD).

#### Role of the facilitator and participatory approach

Attenders and non-attenders at all sites described the group facilitator as a respected source of information and knowledge: “The facilitator gave health information about everything women didn’t know before and then all the women knew about good home care behaviours. They realized what they should do for themselves to improve their health.” (Nepal, non-attender, poor, FGD). The format of the meetings was accessible to illiterate women, as “messages were shared by word of mouth, not by reading and writing.” (India, facilitator, FGD). “Some women who were not actively participating in the discussion in the beginning started to participate little by little, knowing how to address others. We also used participatory tools to guide our discussions.” (Malawi, facilitator, KII). The facilitator was also influential in maintaining the relevance of the group discussions and strategies for both better-off and poor women. For example, awareness-raising strategies and home visits to give advice and information were low-cost and something that poor women could participate in and learn from. Facilitators reminded group members that they should recommend locally relevant actions that could be undertaken by all types of families, and often focused more on poor families: “I think that the women’s group showed an interest in poor families. Poor families were the worst-off and they did not have correct knowledge about good home care practices. Poverty makes poor families weak and makes their mentality weak. In that situation, I saw that facilitators went to poor families and provided them suggestions about good home care practice.” (Nepal, non-attender, better-off, FGD).

#### Learning from each other

Women learned from the facilitator, but at all sites they said that they also learned from each other: “The discussion was like educating each other.” (Malawi, facilitator, KII). Sharing of ideas in the group was discussed as the first step to behaviour change. The group was open to all women and enabled both poor and better-off women to get together, share ideas, and learn from each other: “There is one proverb we have that either people gain knowledge through reading or through experience. Poor families changed their behaviour by seeing whatever others did. The women’s group encouraged them to do good home care practices.” (Nepal, non-attender, KII).

#### Dissemination of health knowledge in the community

All participants felt that increasing knowledge and awareness stimulated behaviour change in group attenders and non-attenders: “Women were interested and asked, what do you do in the women’s group? What do you discuss? So as time went by, they were learning from their friends.” (Malawi, facilitator, KII). “If I learned something, I would not only apply it to myself, but I would also tell others who did not attend the meeting. In this way if someone did not come to the meeting she could know from me or others who attended.” (India, attender, poor, FGD). Group attenders interacted informally with family members, friends and neighbours about how to improve maternal and newborn care behaviours in their daily lives. More formal interactions occurred through implementing strategies to disseminate information about the group and knowledge about good care behaviours. For example, in Malawi, home visits were a strategy implemented by the groups to address water and sanitation concerns: “Because women’s group members were visiting households, things changed and there was good sanitation in the whole village…We noted that if we were to reduce deaths, after going to the toilet we had to wash our hands with soap before we breastfeed the baby. When we wanted to cook food in the kitchen, we washed our hands, washed plates and swept the kitchen. This helped to prevent diseases in our households and all children would not get infected.” (Facilitators, FGD).

Home visits were a popular strategy and had the benefit of being able to reach beyond the immediate networks of women’s group attenders and include poor and better-off families: “The door-to-door visits have helped so much because women did not choose who is a group member and who is not; they visited everyone.” (Malawi, facilitators, FGD). Some participants felt that facilitators and attenders had responded to a need and reached parts of the population not usually accessed: “Previously, due to the lack of proper education, awareness campaigns didn’t reach people. But, later on, health workers, facilitators, and staff from MIRA came to them and made suggestions. It helped to spread awareness everywhere. This is the reason there was behaviour change and women started to accept (recommended) home care practices.” (Nepal, non-attender, poor, changed behaviour). Group attenders felt that this type of dissemination would not have been possible without developing an awareness of how best to support mothers and babies: “Women hardly took iron tablets because they tasted bad or their in-laws did not allow them, but at group meetings we have been told that it is good for us and for our child too. Then our in-laws realised that whatever is told in the group it is good for mothers.”(India, attender, poor, FGD).

The powerful position of the mother-in-law and other family members in overseeing the behaviour of daughters-in-law - particularly newlyweds - was emphasised at Asian sites. In Bangladesh, restrictions preventing women from going outside the home were discussed as significant barriers to accessing information that would be beneficial for their health and the health of their baby. Daughters-in-law felt that they learned a lot if they were allowed to go to the group: “We did not know anything (about maternal and newborn health). We are daughters-in-law; if we had been able to go outside before, we might have known something. But now daughters-in-law can know these things from the comfort of their home. The women’s group came and taught us things, and showed us things would be better if we did these things. So now we know, and we are willing to do them.”(Bangladesh, attender, poor, FGD). Participants in Bangladesh felt that women from better-off families were more restricted in visiting public places and were equally able to learn from groups.

#### Ability to challenge traditional behaviours

In-laws often preferred traditional ways of caring for women and babies: “Family members would think that if I took iron tablets then the baby would grow big, and if my child grows big then I wouldn’t have a normal delivery. I would have a caesarean section. Now these things have changed.” (Bangladesh, facilitator, KII). Women reported feeling able to challenge or convince their own family members and those of others to behave differently after they had been to the group: “(Traditional) practices are diminishing because now we are conscious, we have seen and heard a lot of things. Sometimes we might even engage in disagreements with in-laws. If a mother-in-law suggests something then we suggest a different way of doing things. Like when the mother-in-law still has faith in traditional healers and we opt for a doctor.” (Bangladesh, attender, poor, changed behaviour, SSI). Community support for harmful practices also diminished: “There was the belief that pregnant women should drink traditional medicine to induce labour pains, but this was causing complications. This practice has been reduced in some areas, and in others it has stopped completely.” (Malawi, facilitator, KII).

#### Increased acceptability of recommended practices

In Nepal, Bangladesh, and India, the practice of immediate bathing after birth was particularly challenging to address. Cultural beliefs about birth pollution were related to the practice of bathing with water: “Because the child has *‘*dirt*’* attached to it, if anyone takes the baby on their lap then that person wouldn’t be able to perform Namaz (Islamic prayers). Communities have many superstitions.” (Bangladesh, facilitator, KII). Despite this, study participants felt that behaviour change had occurred through increasing understanding about the effect of bathing on the baby: “When a child is in the mothers’ womb it is warm inside, but when it comes out, the environment is different and the baby may catch cold. Therefore everyone stopped bathing their newborn. This was told at the meeting.” (India, non-attender, better-off, FGD).

In Nepal and Bangladesh, there was some evidence of social prestige in practising behaviours recommended by the group: “Now people follow what they are taught in the meetings. And if someone follows the old superstitions in the village, we tell them to leave those practices as they are from an old era.” (Bangladesh, non-attender, poor, changed behaviour, SSI). Families who practised recommended care behaviours were seen as educated about health issues and forward-thinking: “Because of social prestige, they care about their infants.” (Nepal, attender, poor, FGD).

### Social support

Group attenders were able to talk to others, disseminate information and convince in-laws because their confidence was developed through the meetings. There was some evidence that poor and illiterate women were more apprehensive about the meetings, but their confidence grew once they came and understood the discussion methods: “People who were not educated did not come to meetings earlier. But gradually when they started coming to meetings, their confidence level increased and they started believing in the process. Some newly married women who were educated and came to meetings also brought new ideas and these were discussed in the meeting.” (India, attender, poor, changed behaviour). Participants from all sites felt that the group provided a supportive environment and attenders were proud of what they had achieved together: “We all worked together. I feel like that is how we were able to bring change.” (Nepal, attender, better-off, FGD). The group became respected and influential: “I cannot convince people alone, can I? But now one person teaches many, and they learn things, and we can convince one person with the help of others. When many people try to convince one person then they understand, but you cannot achieve that while you are alone, not everyone is a persuasive person.” (Bangladesh, attender, poor, FGD). Attenders felt supported by other group members: “After coming to the group I think we are all one and we share our problems with each other because we find the solutions for them together.” (India, attender, poor, FGD). There was no evidence from non-attenders about feeling supported by the group, except when they discussed financial support.

### Group and community action

#### Clean delivery kits

In the Asian sites, groups promoted kits to enable clean delivery care. When we discussed increased access to them, participants tended to focus on how the group had increased their knowledge of the need to prevent infection and the benefits of using a kit: “Using a safe delivery kit is safe as mothers will not get an infection” (India, attender, better-off, changed behaviour SSI). With this increase in knowledge there was an increase in demand: “In previous days, different kinds of tools were used to cut a baby’s umbilical cord. Because of those tools, we lost many mothers and babies. But later on, women realized that a kit must be used during delivery. The women’s group made people aware by visiting door-to-door. There was only an increase in the use of kits after the women’s group did that.” (Nepal, non-attender, changed behaviour, SSI). Participants reported an increased demand for kits at the Asian sites, particularly among group attenders: “Those women who had attended the meeting regularly understood the importance of the kit and therefore they demanded it from Auxiliary Nurse Midwives while delivering their child.” (India, facilitator, KII). The availability of kits was particularly beneficial to women who were restricted from going outside their homes. In Bangladesh, group attenders “took the kits from facilitators and gave them to pregnant women.” (Bangladesh, attender, poor, FGD). Participants in Bangladesh and India felt that the increased demand and utilisation of kits was indicative of advance preparation for a delivery: “Now people have become cautious these days, they keep the kit at home in advance.” (Bangladesh, non-attender, better-off, FGD). Groups in Nepal made and sold kits at lower cost than available brands and poor women in India and Nepal made the kits themselves: “If poor women couldn’t buy the kit, they made it by themselves by boiling the blade in water, using clean clothes and bringing thread and soap etc.” (Nepal, non-attender, better-off, FGD). Participants felt that the role of a kit in preventing illness was particularly important for poor women: “Poor people know that in case of any emergency they don’t have enough resources to deal with it. Therefore they prefer to be prepared for these things. Preparing safe delivery kits is one example of this.” (India, attender, better-off, changed behaviour, SSI). Women in Malawi did not use or promote clean delivery kits, but participants elsewhere felt there had been increased demand for health services as a result of the group intervention.

#### Financial support

The strategies developed by women’s groups also enabled some financial support to women. The vegetable gardens in Malawi helped them to eat better during pregnancy, and any surplus was sold and the income put in a fund: “This strategy really helped because a baby needs to eat a variety of food to grow. People used this money to assist pregnant women so that they could eat what they felt like eating. They could take the money and buy anything, which made the women and unborn baby happy.” (Malawi, facilitator KII). Funds created by groups in Bangladesh, Malawi, and Nepal were used to lend money to women. Both poor and better-off women accessed them: “If we are not able to pay for nutritious foods in the period of pregnancy and delivery we sometimes use money from the women’s group fund, and give money back later when we can. We use the fund turn by turn.” (Nepal, non-attender, better-off, FGD). Although the funds were meant to have a maternal and newborn health focus, they were also used for other small expenses, and women could repay at very low interest when they were able. “In the past someone might have a shortage of money, but now we have overcome even that, now that our group has an emergency fund. We can take money from there.” (Bangladesh, attender, poor, FGD). Some groups raised money for non-attenders who had not invested but required help. One non-attender told us: “Although I am not involved in the group, I can get lots of support from it. Other women have also received up to 5000 rupees from the women’s group.” (Nepal, non-attender, poor, FGD). The women’s group network was a source of social and financial support for women, irrespective of attendance, in all sites: "If someone has a problem we could discuss it as a group and give her part of the money to help. When someone is sick we can agree among ourselves to go and see her in the hospital using the group fund (Malawi, facilitator, FGD). At all sites, funds helped group attenders feel more confident: “We can all make decisions. We have money so we have a different kind of strength.” (Bangladesh, attender, better-off, FGD).

## Discussion

Inequalities in childhood mortality are a persistent problem [4] and little is known about what works to reduce them [7–9]. Contrary to Tudor Hart’s Inverse Care Law [30], participatory women’s groups have been shown to equitably reduce neonatal mortality and improve health behaviors across all social strata [20]. Our present qualitative study contributes to the literature by analysing how these equitable impacts were achieved. We found that the social process of learning and action –which led to increased knowledge, confidence to act, and acceptability of recommended practices- was crucial. The PLA approach increased knowledge in group attenders and community members, and groups enabled an increased understanding of health promotion messages disseminated by other organisations and the media. This catalysed an acceptance of ‘new’ and healthy behaviours as group attenders interacted with the community. The participatory process gave women confidence in their knowledge and they felt supported by the group, which in turn helped them convince family members of the benefits of healthy behaviours. Community support for some harmful practices diminished and some recommended practices, such as delayed bathing of the newborn, became more acceptable. There is evidence that the group became a respected and influential source of knowledge and that there was social prestige in practising the recommended behaviours.

Participants felt that the equal impact of the intervention on behaviours among poor and better-off women was due to a knowledge deficit, irrespective of socioeconomic position. In Bangladesh, better-off women were less able to leave their homes and interaction with group members was an important source of knowledge and support. The style of learning enabled women from all strata to understand the discussions and develop confidence. Facilitators encouraged the involvement of poor women and the development of strategies that met the needs of poor as well as better-off women. The groups promoted healthy home care behaviours that poor and better-off women could implement. When changes were too difficult for poor women to enact, groups provided a widened social network, access to a fund, and other resources like clean home delivery kits. If recommended behaviours were out of reach for poor households, we would probably not have seen equitable effects on poor and better-off groups.

### Links to theory

#### Social learning and social cognitive theory

Our findings support theories that emphasise the mediating role of social-cognitive factors (behavioural beliefs, perceived behavioural control, social norms) in the relationship between socioeconomic position and health behaviour [31, 32]. Knowledge deficits and constraining community and family norms were significant barriers to healthy behaviours across socioeconomic strata in our study population. The intervention was able to influence behavioural beliefs and social norms so that recommended practices became more acceptable. The PLA process gave women confidence to change their behaviour and influence others to do the same. Bandura’s social learning theory emphasises the importance of social interaction for behaviour change. We found that the facilitator and intervention design shaped the process of social interaction in such a way that the intervention was inclusive of all socioeconomic strata.

The PLA process increased group attenders’ confidence to challenge powerful norms and convince community members about the benefits of healthy practices. These findings support theories that emphasise the importance of social change for improvements in maternal and newborn health. Our findings suggest that the women’s group intervention addresses some structural and agency constraints to reducing health inequalities. Power structures were similar in poor and better-off households, with the exception of Bangladesh where better-off women were perhaps more constrained. The development literature suggests that the PLA approach facilitates dialogue and exchange of ideas and experiences, which enable groups and communities to become critically consciousness of the constraints to health improvement, and this motivates them to change their situation [11, 14]. This process enables communities and individuals to engage with power structures and recognises the role of agency.

#### Social networks and context

Despite obvious contextual differences, for example the restrictions on women in South Asian sites as compared to Malawi, the mechanisms stimulating behaviour change in the four research sites were similar. The participatory nature of the intervention allowed adaption to context, with women and communities identifying local problems and ways to address them.

We found that women’s groups were effective in strengthening or creating social networks, which enabled women to access a broader basis of social support. Strengthened reciprocity and social networks between group attenders and within communities enabled behaviour change in maternal and newborn health.

Although study contexts were culturally different, studies were all conducted in rural areas. Neighbours knew one another and communities arguably had a strong sense of social control. The women’s group intervention took advantage of this to create community pressure for behaviour change. In poor, urban, and perhaps more anonymous communities, the intervention has not been as effective at changing behaviours [33].

Finally, the intervention was implemented in a context where families reported a lack of knowledge about healthy practices. Our findings suggest that the PLA approach is most likely to be equally effective across socioeconomic strata for issues about which there is a knowledge deficit and for which simple changes in the home can be implemented. Facilitators and group attenders were able to offer advice about affordable actions that were contextually relevant and acceptable. This advice, combined with increased social support, confidence and acceptability of healthy behaviours, enabled families to change their behaviour. If healthy behaviours had incurred financial costs to families, the intervention might not have been so effective across socioeconomic strata.

### Limitations

The way that the results were reported made it difficult to distinguish between spontaneous responses and those provoked by discussion of process evaluation findings. We were aware of this potential limitation, and checked translated transcripts, which revealed that respondents very often spontaneously raised similar issues. We feel, therefore, that this is not a substantial limitation of our study.

Leadership by researchers who were directly involved in the trials might have biased data collection and reporting. We felt that the disadvantages of this approach were outweighed by the advantages of having an experienced researcher who understood the intervention and process evaluation data.

We explored and expanded a linear model of how women’s groups enabled behaviour change in poor and better-off women. The causal structure of these mechanisms is arguably more complex and expansive than presented here. Mechanisms are a sequence of linked activated entities that occur repeatedly if the context is enabling [34], and it was often difficult for participants to consider them separately. Mechanisms were usually considered as an inseparable synergy of ways that the groups interacted with the community to change behaviours.

### Implications

Randomised controlled trials have been criticised for inadequately exploring intervention effects on sub-groups and the pathways through which interventions influence health outcomes. This can restrict the development of theory about how they work and limit analysis of their external validity [35, 36]. Sub-group analysis is particularly important, as there is an urgent need to know which interventions reach the poorest, and to understand how they work.

Interestingly, the PLA approach may work particularly well in contexts that are usually considered as challenging from a public health perspective: in communities with a lack of knowledge about healthy practices across all socioeconomic groups, with restrictive norms and a strong sense of social control. Because the approach involved all socioeconomic strata and uses a social process of learning and action, these contexts may paradoxically create an opportunity for effect. The PLA approach is perhaps less effective in more anonymous settings.

## Conclusions

Our analysis shows that the social process of learning and action -which led to increased knowledge, confidence to act, and acceptability of recommended practices- was key to ensuring effective behaviour change. The equitable behavioural effects were facilitated by the accessibility, relevance, and engaging format of the intervention across socioeconomic groups, and by reaching-out to parts of the population usually not accessed. The PLA approach was effective in improving health behaviours across all socioeconomic strata in rural communities for issues around which there was a knowledge deficit and where simple changes in the home could be implemented.

## Abbreviations

FGD: Focus group discussions
KII: Key informant interview
PLA: Participatory learning and action
SSI: Semi-structured Interview
TBA: Traditional birth attendant
